# Gallbladder perforation during peroral holmium laser lithotripsy

**DOI:** 10.1055/a-2868-7854

**Published:** 2026-05-21

**Authors:** Rui Zhu, Hui Long, Yue Zhu, Qiu Zhao, Hongling Wang, Fan Wang

**Affiliations:** 1 Department of GastroenterologyTianyou Hospital Affiliated to Wuhan University of Science and TechnologyWuhanChina; 2 Department of Gastroenterology 89674 Wuhan University Zhongnan HospitalWuhanChina

**Fig. 1:**
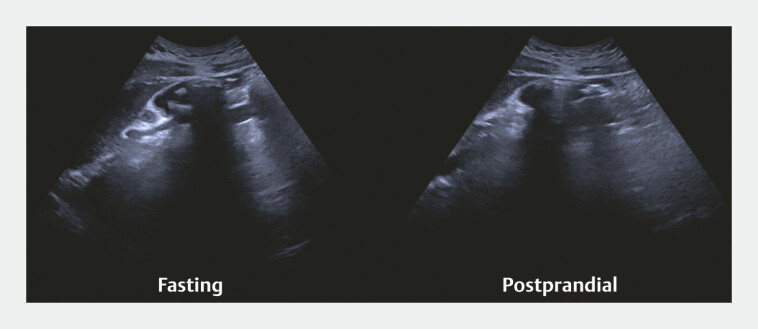
Abdominal ultrasonography indicated multiple large stones in the gallbladder and an ejection fraction of 80% on a fatty meal test.

**Fig. 2:**
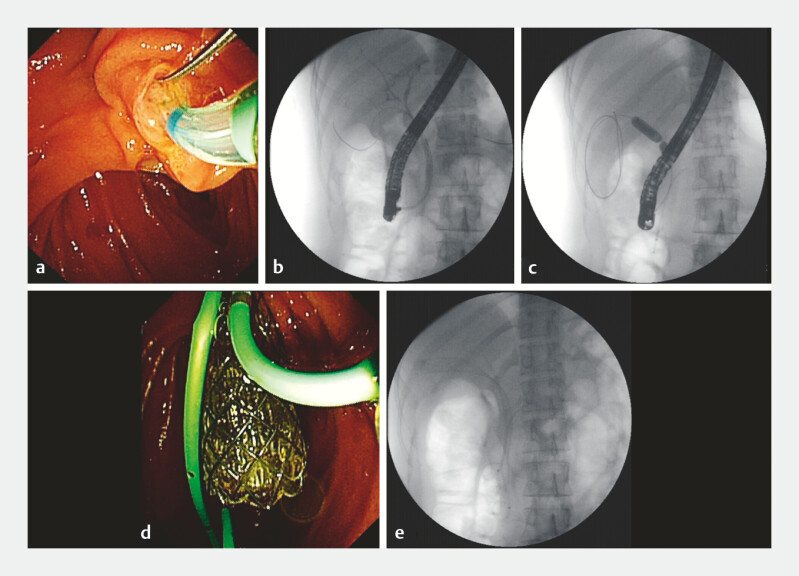
**a–c** Guidewire insertion into the gallbladder using ERCP techniques and **d, e** stent placement across the papilla bridging the duodenum and gallbladder. ERCP, endoscopic retrograde cholangiopancreatography.

**Fig. 3:**
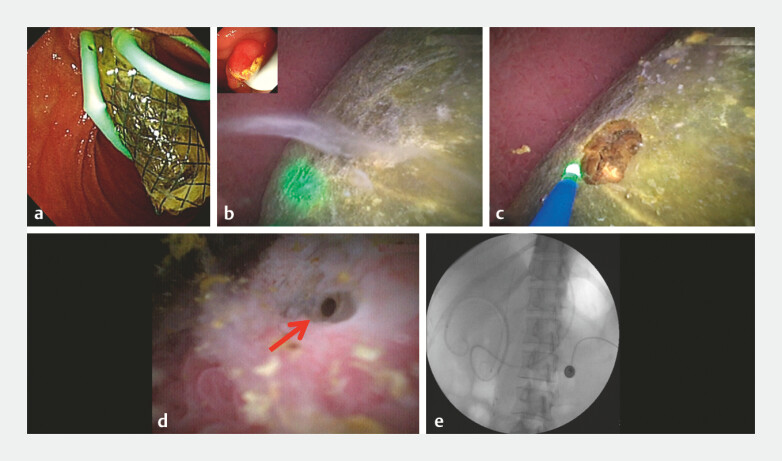
**a, b** Peroral choledochoscope detection of multiple large stones in the gallbladder. **c** Holmium laser lithotripsy. **d** Gallbladder perforation occurring during the lithotripsy (arrow). **e** Nasobiliary tube placement for continuous negative-pressure bile drainage.

**Fig. 4:**
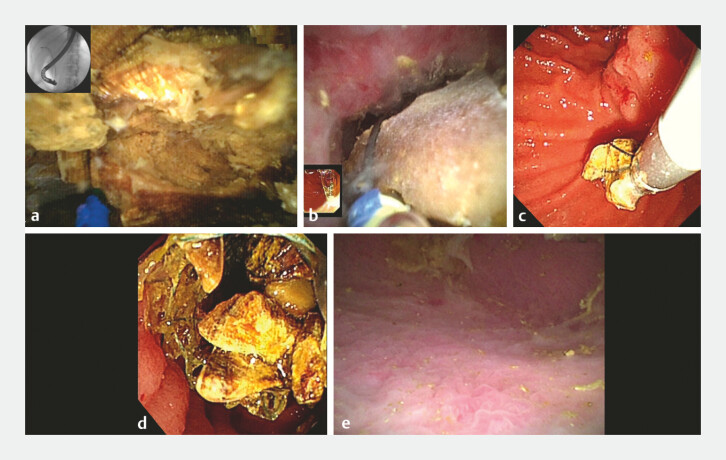
**a** The second lithotripsy session, **b–d** stone extraction, and **e** complete healing of the perforation in the gallbladder after 1 week.

A 26-year-old woman presented with intermittent right upper quadrant abdominal pain for 6 months. Abdominal ultrasonography revealed multiple stones in the gallbladder (largest size: 22 × 9 mm; wall thickness: 2 mm) and an ejection fraction of 80% on a fatty meal test ( Fig. 1 ). Given the patient’s strong preference for gallbladder preservation, peroral gallbladder-preserving cholelithotomy using a choledochoscope (eyeMAX, 9-Fr, Micro-Tech, China) was planned 1 . During the procedure, a guidewire was inserted into the gallbladder using endoscopic retrograde cholangiopancreatography techniques, and a fully covered metal stent (120 × 10 mm, Micro-Tech, China) was implanted across the papilla, bridging the duodenum and gallbladder ( Fig. 2 ). One week later, the choledochoscope was easily advanced through the stent into the gallbladder, where multiple large stones were identified ( Fig. 3 ). Subsequently, holmium laser lithotripsy was performed ( Video 1 ). However, the laser inadvertently penetrated the gallbladder wall, resulting in acute gallbladder perforation. A nasobiliary tube was immediately placed for bile drainage, and subsequent continuous negative-pressure drainage was applied. After 1 week, choledochoscopy confirmed the complete healing of the perforation. A second lithotripsy session was performed for the residual stones, followed by complete stone extraction ( Fig. 4 ). The patient was discharged shortly thereafter and remained asymptomatic during the 7-month follow-up. To the best of our knowledge, this is the first reported endoscopic detection and management of gallbladder perforation occurring during peroral holmium laser lithotripsy.

Gallbladder perforation during peroral holmium laser lithotripsy.Video 1

Endoscopy_UCTN_Code_CPL_1AK_2AF
